# Does the primary tumour location affect the prognosis of patients with colorectal cancer peritoneal metastases treated with cytoreductive surgery and hyperthermic intraperitoneal chemotherapy?

**DOI:** 10.1186/s12957-021-02374-4

**Published:** 2021-08-26

**Authors:** Haipeng Chen, Sicheng Zhou, Jianjun Bi, Qiang Feng, Zheng Jiang, Jianping Xu, Wei Pei, Jianwei Liang, Zhixiang Zhou, Xishan Wang

**Affiliations:** 1 Department of Colorectal Surgery, National Cancer Center/National Clinical Research Center for Cancer/Cancer Hospital, Chinese Academy of Medical Sciences and Peking Union Medical College, Beijing, 100021 China; 2 Department of Medical Oncology, National Cancer Center/National Clinical Research Center for Cancer/Cancer Hospital, Chinese Academy of Medical Sciences and Peking Union Medical College, Beijing, 100021 China

**Keywords:** Cytoreductive surgery, Hyperthermic intraperitoneal chemotherapy, Primary tumour location, Prognosis

## Abstract

**Background:**

The impact of primary tumour location on the prognosis of patients with peritoneal metastasis (PM) arising from colorectal cancer (CRC) after cytoreductive surgery (CRS) and hyperthermic intraperitoneal chemotherapy (HIPEC) is rarely discussed, and the evidence is still limited.

**Methods:**

Patients with PM arising from CRC treated with CRS and HIPEC at the China National Cancer Center and Huanxing Cancer Hospital between June 2017 and June 2019 were systematically reviewed. Clinical characteristics, pathological features, perioperative parameters, and prognostic data were collected and analysed.

**Results:**

A total of 70 patients were divided into two groups according to either colonic or rectal origin (18 patients in the rectum group and 52 patients in the colon group). Patients with PM of a colonic origin were more likely to develop grade 3–4 postoperative complications after CRS+HIPEC (38.9% vs 19.2%, *P* = 0.094), but this difference was not statistically significant. Patients with colon cancer had a longer median overall survival (OS) than patients with rectal cancer (27.0 vs 15.0 months, *P* = 0.011). In the multivariate analysis, the independent prognostic factors of reduced OS were a rectal origin (HR 2.15, 95% CI 1.15–4.93, *P* = 0.035) and incomplete cytoreduction (HR 1.99, 95% CI 1.06–4.17, *P* = 0.047).

**Conclusion:**

CRS is a complex and potentially life-threatening procedure, and we suggest that the indications for CRS+HIPEC in patients with PM of rectal origin be more restrictive and that clinicians approach these cases with caution.

## Introduction

Colorectal cancer (CRC) is one of the most common malignant tumours in the world, and its morbidity and mortality rank third and fourth, respectively [1–3]. Among patients with CRC, 5–15% have synchronous peritoneal metastasis (PM), and the incidence of metachronous PM is as high as 20 to 50% [4]. PM arising from CRC is an indicator of terminal stage disease and carries a very poor prognosis. In the past, palliative surgery and systemic chemotherapy were mostly adopted, but the therapeutic effect was poor, and the median survival time was only 5 to 7 months [5]. Currently, cytoreductive surgery (CRS) combined with hyperthermic intraperitoneal chemotherapy (HIPEC) has shown good clinical efficacy in the comprehensive treatment of various malignant peritoneal diseases, including CRC, ovarian cancer, and appendiceal mucous adenocarcinoma, and it has been considered a standard therapy for prolonging the survival of patients with PM arising from CRC [6–10].

At present, most of the extant literature has demonstrated that a high peritoneal cancer index (PCI), incomplete cytoreduction, young age, lymphovascular invasion, and postoperative complications are poor prognostic factors after CRS+HIPEC [11–15]. It has been well established that different primary tumour locations have different biological behaviours and prognosis [16–19]. However, previous studies have only compared the prognostic differences between the colonic origin and rectal origin of the primary tumour; the impact of the primary tumour location on the prognosis of patients with PM arising from CRC after CRS+HIPEC is rarely discussed, and clinical evidence remains scarce [20]. It is highly desirable to optimize patient selection to include only those who are most likely to benefit from this complex and potentially life-threatening procedure. Therefore, the aim of this study was to explore the impact of primary tumour location according to colon or rectal origin on the prognosis of patients with PM arising from CRC treated with CRS+HIPEC in our institution.

## Methods

### Study design and patients

The study protocol was approved by the Ethics Committee of the Cancer Hospital, Chinese Academy of Medical Sciences (NCC2017-YZ-026, October 17, 2017). The data of all patients with synchronous or metachronous PM arising from CRC who underwent CRS with HIPEC at the National Cancer Center and Huanxing Cancer Hospital were retrospectively obtained from a prospectively maintained database between June 2017 and June 2019. The inclusion criteria of this study were as follows: (1) patients with PM of a colonic or rectal origin, (2) pathologically confirmed PM after operation, and (3) age between 18 and 75 years. The exclusion criteria were as follows: (1) complications with the liver, lung, or other sites of distant metastasis; (2) history of other malignant tumours; and (3) malignant tumour of appendix origin. According to the location of the primary tumour, all enrolled patients were divided into a colon group (*n* = 52) and a rectal group (*n* = 18). The colon was regarded as the caecum, ascending colon, transverse colon descending colon, and sigmoid colon, while the rectum was regarded as the intestinal canal below 15 cm from the anal margin.

Demographic and clinical variables, as well as perioperative and long-term survival outcomes, were collected and compared. All enrolled patients underwent a routine preoperative evaluation, which included laboratory examinations, abdominal contrast-enhanced computed tomography, pelvic magnetic resonance imaging, and fluorodeoxyglucose positron emission tomography, to assess their general condition. According to the Sugarbaker/Jacquet classification, peritoneal disease burden and the completeness of cytoreduction were assessed using the PCI and completeness of cytoreduction (CC) score, respectively [21, 22]. All postoperative complications were graded using the Clavien-Dindo classification according to the treatment received [23].

### Surgical technique

The surgical techniques adopted at our institution have been previously described [9, 21]. Briefly, three outflow drains and one inflow drain were routinely placed in the abdomen in preparation for HIPEC. HIPEC was administered in a closed fashion, with oxaliplatin (200 mg/m^2^) and raltitrexed (3 mg/m^2^), combined with or without lobaplatin (50 mg/m^2^). Then, patients were treated with a mixed solution of chemotherapy agents and 3 l of saline solution infused into the abdominal and pelvic cavity for 60 min at 42–43 °C. Next, two additional HIPEC procedures were performed in the ward on the second and fourth days after surgery in both groups. Furthermore, two surgical specialists with more than 20 years of experience in gastrointestinal surgery performed the operations at the two centres, and the HIPEC technique and postoperative treatment were identical.

### Statistical analysis

Data between two groups were analysed with SPSS 24.0 software (IBM, Armonk, NY, USA). Categorical data are expressed as percentages and were compared using the χ^2^ test or Fisher’s exact test as appropriate. Continuous data are expressed as the mean ± standard deviation and were compared using Student’s *t*-test and the Mann-Whitney *U* test for independent values with normally and nonnormally distributed values, respectively. Overall survival (OS) was defined as the time from surgery to the time of death from any cause or July 31, 2020, whichever came first. The Kaplan-Meier method and log-rank test were utilized to evaluate associations between individual factors and OS. Variables found to be significant (*P* value < 0.20) in the univariate analysis were incorporated into the multivariate analysis to identify independent predictors of OS. A *P* value < 0.05 was considered statistically significant.

## Results

### Demographic and clinical variables

A total of 70 patients with PM of CRC origin who underwent CRS+HIPEC were included in the present study. Of these patients, 18 (25.7%) had rectal cancer and 52 (74.3%) had colon cancer. The mean age of all patients was 54.5 years, and the majority (55.7%) of patients in the study were male. Patients were well balanced across the two groups in terms of age, sex, body mass index (BMI), preoperative comorbidity, preoperative chemotherapy, presentation of PM, preoperative CEA level, preoperative CA19-9 level, histology, tumour grade, adjuvant chemotherapy, BRAF status, and MSI (*P* > 0.05) (Table 1).

**Table 1:** Patient characteristics

Characteristics	Total (*n* = 70)	Rectum (*n* = 18)	Colon (*n* = 52)	*P*
Age at operation (years, mean ± SD)	54.5 ± 11.6	52.7 ± 12.2	55.2 ± 11.6	0.451
Sex (%)				0.571
Male	39 (55.7)	9 (50.0)	30 (57.7)	
Female	31 (44.3)	9 (50.0)	22 (42.3)	
Body mass index (kg/m^2^, mean ± SD)	22.7 ± 3.6	23.8 ± 3.9	22.8 ± 3.5	0.665
Comorbidity	18 (25.7)	4 (22.2)	14 (26.9)	0.936
Hypertension	10 (14.3)	2 (11.1)	8 (15.4)	
Diabetes	6 (8.6)	2 (11.1)	4 (7.7)	
Coronary heart disease	2 (2.9)	1 (5.5)	1 (1.9)	
Arrhythmia	4 (5.7)	0 (0)	4 (7.7)	
Others	6 (8.6)	1 (5.5)	5 (9.6)	
Preoperative chemotherapy (%)				0.477
Presence	30 (42.9)	9 (50.0)	21 (40.4)	
Absence	40 (57.1)	9 (50.0)	31 (59.6)	
Presentation of PM (%)				0.118
Synchronous	42 (60.0)	8 (44.4)	34 (65.4)	
Metachronous	28 (40.0)	10 (55.6)	18 (34.6)	
T stage				0.954
T1–T2	5 (11.9)	1 (12.5)	4 (11.8)	
T3–T4	37 (88.1)	7 (87.5)	30 (88.2)	
N stage				0.482
N0	2 (4.8)	0 (0)	2 (5.9)	
N1–N2	40 (95.2)	8 (100.0)	32 (94.1)	
Preoperative CEA level (ng, mean ± SD)	31.9 ± 61.5	16.6 ± 27.2	37.2 ± 69.8	0.290
Preoperative CA19-9 level (ng, mean ± SD)	75.4 ± 93.3	69.2 ± 112.4	77.5 ± 88.4	0.780
Histology (%)				0.275
Adenocarcinoma	43 (61.4)	13 (72.2)	30 (57.7)	
Mucinous	27 (38.6)	5 (27.8)	22 (42.3)	
Tumour grade				0.370
Moderate	26 (37.1)	8 (44.4)	17 (32.7)	
Poor	44 (62.9)	10 (55.6)	35 (67.3)	
Adjuvant chemotherapy				0.924
Presence	55 (78.6)	14 (77.8)	41 (78.8)	
Absence	15 (21.4)	4 (22.2)	11 (21.2)	
BRAF status				0.809
Mutation	13 (18.6)	3 (16.7)	10 (19.2)	
No mutation	57 (81.4)	15 (83.3)	42 (80.8)	
MSI				0.961
MSI-H	8 (11.4)	2 (11.1)	6 (11.5)	
MSS	62 (88.6)	16 (88.9)	46 (88.5)	

Note: *SD* standard deviation, *PM* standard peritoneal metastasis

### Operative and perioperative data

The operative details and postoperative courses are listed in Table 2. The mean PCI of all enrolled patients was 11.1, and complete cytoreduction (CC 0–1) was achieved in most patients (68.6%). Patients in both groups had comparable mean operative times (255.5 vs 257.4 min, *P* = 0.922) and estimated blood loss (98.9 vs 130.2 ml, *P* = 0.301). Patients in the rectal group were more likely to undergo colostomy or ileostomy (66.7% vs 30.8%, *P* = 0.007) during the operation. Compared with patients in the colon group, patients in the rectum group were more likely to develop grade 3–4 postoperative complications (38.9% vs 19.2%, *P* = 0.094), but this difference was not statistically significant. Ileus (7.1%) and pelvic cavity abscesses (7.1%) were the most common postoperative complications, followed by anastomotic leakage (5.7%), wound infection (2.9%), pneumonia (5.4%), pleural effusion (1.4%), cardiac arrhythmia (1.4%), urinary retention (1.4%), and rectovaginal leakage (1.4%). Two patients (2.9%) required revision surgery due to extensive pelvic cavity abscesses and postoperative bleeding.

**Table 2:** Operative and perioperative data

Characteristic	Total (*n* = 70)	Rectum (*n* = 18)	Colon (*n* = 52)	*P*
Operative method				0.538
Laparoscopic surgery	14 (20.0)	5 (27.8)	9 (17.3)	
Open surgery	56 (80.0)	13 (72.2)	43 (82.7)	
HIPEC regimen				0.900
Lobaplatin + oxaliplatin + raltitrexed	32 (45.7)	8 (44.4)	24 (46.2)	
Oxaliplatin + raltitrexed	38 (54.3)	10 (55.6)	28 (53.8)	
Colostomy or ileostomy				0.007
Presence	28 (40.0)	12 (66.7)	16 (30.8)	
Absence	42 (60.0)	6 (33.3)	36 (69.2)	
PCI score (mean ± SD)	11.1 ± 6.0	11.7 ± 6.9	10.8 ± 5.8	0.600
Presence of ascites				0.693
Presence	30 (42.9)	7 (38.9)	23 (44.2)	
Absence	40 (57.1)	11 (61.1)	29 (55.8)	
CC score				0.168
CC 0–1	48 (68.6)	10 (55.5)	38 (73.1)	
CC 2–3	22 (31.4)	8 (44.5)	14 (26.9)	
Operative time, min (mean ± SD)	256.9 ± 66.0	255.5 ± 83.5	257.4 ± 60.4	0.922
Estimated blood loss, ml (mean ± SD)	122.1 ± 109.2	98.9 ± 82.9	130.2 ± 117.5	0.301
Postoperative complications (grades III, IV)	17 (24.3)	7 (38.9)	10 (19.2)	0.094
Postoperative bleeding	2 (2.9)	1 (5.6)	1 (1.9)	
Anastomotic leakage	4 (5.7)	2 (11.1)	2 (3.8)	
Pelvic cavity abscess	5 (7.1)	2 (11.1)	3 (5.8)	
Ileus	5 (7.1)	2 (11.1)	3 (5.8)	
Pneumonia	1 (1.4)	1 (5.6)	0 (0)	
Pleural effusion	1 (1.4)	1 (5.6)	0 (0)	
Cardiac arrhythmia	1(1.4)	0 (0)	1 (1.9)	
Wound infection	2 (2.9)	1 (5.6)	1 (1.9)	
Urinary retention	1 (1.4)	0 (0)	1 (1.9)	
Rectovaginal leakage	1 (1.4)	0 (0)	1 (1.9)	
Postoperative hospital stay, days (mean ± SD)	14.6 ± 5.3	15.4 ± 4.7	14.3 ± 5.6	0.380
Re-operation	2 (2.9)	1 (5.6)	1 (5.6)	1.000
Mortality	0 (0)	0 (0)	0 (0)	–

Note: *HIPEC* standard hyperthermic intraperitoneal chemotherapy, *PCI* standard peritoneal cancer index, *CC* standard complete cytoreduction

### Overall survival

**Fig. 1:**
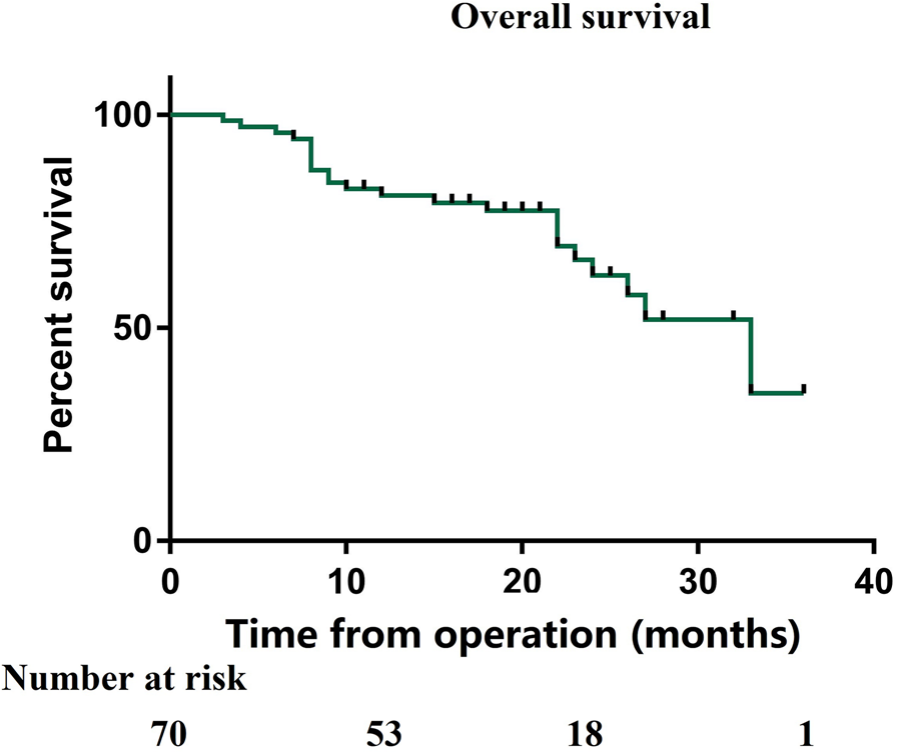
Overall survival of the entire cohort

**Fig. 2:**
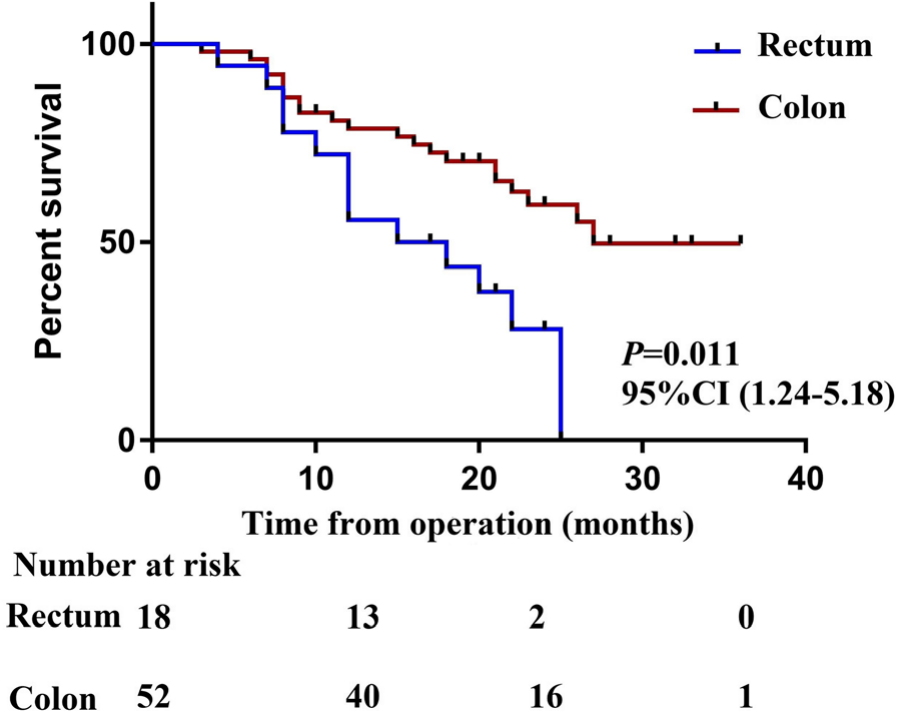
The median OS for those with colon cancer vs those with rectal cancer

**Fig. 3:**
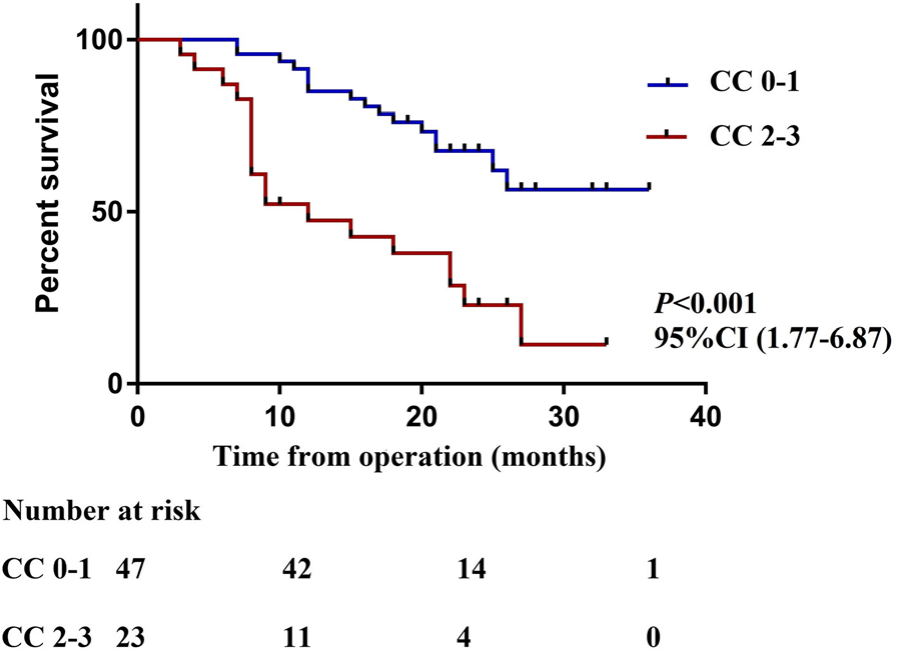
The median OS from CRS+HIPEC in patients undergoing incomplete cytoreduction vs complete cytoreduction

The median estimated follow-up period from CRS/HIPEC for the study population was 28 months. The median survival period for all patients was 25 months, and the estimated 1-, 2- and 3-year OS rates for the entire cohort were 72.6%, 51.4%, and 40.1%, respectively (Fig. 1). The median OS for those with colon cancer was 27 months compared with 15 months for those with rectal cancer (*P* = 0.011) (Fig. 2). The median OS from CRS+HIPEC in patients undergoing incomplete cytoreduction (CC 2–3) was 12 months, while the median OS was not reached in patients undergoing complete cytoreduction (CC 0–1) (Fig. 3). Variables with *P* < 0.20 in the univariate regression analysis, such as increasing PCI (HR 1.09, 95% CI 1.03–1.14, *P* = 0.002), rectal origin (HR 2.54, 95% CI 1.24–5.18, *P* = 0.011), incomplete cytoreduction (HR 3.49, 95% CI 1.77–6.87, *P* < 0.001), and HIPEC regimen (HR 0.63, 95% CI 0.31–1.28, *P* = 0.199), were included in the multivariate analysis. In the multivariate analysis, independent prognostic factors of reduced OS were rectal origin (HR 2.15, 95% CI 1.15–4.93, *P* = 0.035) and incomplete cytoreduction (HR 1.99, 95% CI 1.06–4.17, *P* = 0.047) (Table 3).

**Table 3:** Univariate analysis and multivariate analysis

Variables	Overall survival			
	Univariate analysis		Multivariate analysis	
	HR (95% CI)	*P*	HR (95% CI)	*P*
Sex: male/female	1.32 (0.66–2.64)	0.434		
Age at operation	1.02 (0.98–1.05)	0.283		
Preoperative chemotherapy (no/yes)	1.22 (0.53–2.80)	0.635		
Synchronous/metachronous	1.46 (0.73–2.93)	0.288		
Site of original (rectum/colon)	2.54 (1.24–5.18)	0.011	2.15 (1.15–4.93)	0.035
Histology (mucinous/adenocarcinoma)	1.53 (0.78–3.00)	0.215		
Preoperative CEA level	1.00 (0.99–1.00)	0.279		
Preoperative CA19-9 level	1.00 (0.99–1.00)	0.247		
HIPEC regimen (lobaplatin/non-lobaplatin)	0.63 (0.31–1.28)	0.199	1.39 (0.65–2.94)	0.394
Presence of ascites (yes/no)	1.33 (0.68–2.60)	0.410		
PCI score	1.09 (1.03–1.14)	0.002	1.05 (0.98–1.12)	0.140
CC score (2–3/0–1)	3.49 (1.77–6.87)	< 0.001	1.99 (1.06–4.17)	0.047
Grade 3–4 postoperative complication (no/yes)	1.63 (0.77–3.42)	0.201		
Leukopenia (no/yes)	0.67 (0.28–1.63)	0.382		
Neutropenia (no/yes)	0.80 (0.33–1.94)	0.626		
Thrombocytopenia (no/yes)	0.49 (0.15–1.63)	0.245		
BRAF status (mutation/no mutation)	1.58 (0.79–3.13)	0.266		
MSI (MSS/MSI-H)	1.27 (0.61–2.73)	0.573		
Adjuvant therapy (yes/no)	0.76 (0.35–1.65)	0.489		
Colostomy or ileostomy (yes/no)	1.39 (0.71–2.74)	0.334		

## Discussion

Primary tumour location is recognized as an important prognostic factor for metastatic CRC, and it is also a selection factor for the administration of different targeted medicines [16–19]. However, the impact of primary tumour location on the prognosis of CRC patients undergoing CRS+HIPEC due to PM is rarely discussed, so the available evidence remains limited [20]. Therefore, we conducted this study to elucidate the differences in different primary tumour locations among patients with PM arising from CRC and focused on the significant impacts of these differences on perioperative outcomes and long-term prognosis.

In the present study, 70 enrolled patients were divided into two groups according to the origin of the primary tumour: the colon group (52 patients) and the rectal group (18 patients). The average age of the patients included in this study was only 54.5 years old, and only 25.7% of patients had comorbidities before surgery. This may be due to the aggressive tumour behaviour observed in young patients; these tumours show high invasiveness and a predilection towards distant metastases in regions such as the peritoneum in this population. Our results revealed that patients with colon cancer-derived PM had a longer median survival after CRS+HIPEC (27.0 vs 15.0 months, *P* = 0.011), and primary tumour location remained an independent predictor of OS (HR 2.15, 95% CI 1.15–4.93, *P* = 0.035). In 2018, Tonello et al. [17] published a paper in which they analysed survival in patients with colorectal PM treated with CRS+HIPEC and reported that PM of a rectal origin was associated with worse long-term survival outcomes than PM of a colonic origin (median OS 47.8 vs 22.0 months, *P* = 0.008). Similarly, Da Silva et al. [16] also demonstrated that the median survival in patients with PM of a colonic origin was significantly better than that in patients with PM of a rectal origin (35.0 vs 17.0 months). The above research results are basically consistent with our findings.

Several theories have been proposed to explain this difference in terms of the prognosis of PM of a rectal origin. Anatomically, rectal tumours are located in a narrow pelvic cavity, which makes resection of the primary tumour and pelvic peritoneal metastasis difficult; therefore, achieving complete cytoreduction is a challenge, and the possibility of a residual tumour is increased [24]. Low-middle rectal cancer (under the peritoneum) increases the risk of perforating the rectal wall, which is thicker than the colon wall; the thickness of the rectal wall is the reason underlying the more biologically aggressive disease characteristics observed in this population [16]. Finally, patients with peritoneal metastases originating from rectal cancer are more likely to develop postoperative complications, and the occurrence of complications negatively affects the overall condition of the patients, as well as subsequent adjuvant treatment, and thus has an impact on prognosis. However, the above mechanisms are limited to only a theoretical level; additional studies are needed in the future to further explain the differences in the prognosis of patients with PM arising from different sites of origin at the genetic level.

Close attention has been given to the morbidity and mortality associated with the CRS+HIPEC procedure. Our institution confirmed that the grade 3–4 morbidity and mortality rates after CRS+HIPEC were 24.3% and 0%, respectively, which is basically consistent with the results reported by international centres [9, 14, 25–27]. Notably, we also found that patients with PM of a rectal origin were more likely to develop grade 3–4 postoperative complications after CRS+HIPEC than patients with PM of a colonic origin (38.9% vs 19.2%, *P* = 0.094), but this difference was not statistically significant. CRS is an originally complex and potentially life-threatening procedure. Due to the special anatomical location of rectal tumours, the narrow operating space further increases the difficulty of CRS.

The limitations of this study are those inherent to a single institution with a limited sample size, which may underlie some of the differences observed between the rectal group and the colon group. Second, this study was also limited by its retrospective nature, which makes it difficult to control for bias and confounders. Therefore, we recommend that clinicians exert caution when making any definitive conclusions. Multicentre prospective randomized controlled studies are required to further verify our results.

## Conclusion

Patients with PM of a rectal origin were more likely to develop postoperative complications after CRS+HIPEC, which is indicative of a poor prognosis. We suggest that the indications for CRS+HIPEC in patients with PM of rectal origin should be more restrictive and cautious.

## Data Availability

The datasets generated and/or analysed during the current study are not publicly available because the data are confidential patient data but are available from the corresponding author upon reasonable request.

## Abbreviations

PM: Peritoneal metastasis
CRC: Colorectal cancer
CRS: Cytoreductive surgery
HIPEC: Hyperthermic intraperitoneal chemotherapy
PCI: Peritoneal cancer index
CC: Completeness of cytoreduction
OS: Overall survival
BMI: Body mass index
